# A Mathematical Approach to Correlating Objective Spectro-Temporal Features of Non-linguistic Sounds With Their Subjective Perceptions in Humans

**DOI:** 10.3389/fnins.2019.00794

**Published:** 2019-07-31

**Authors:** Thomas Burns, Ramesh Rajan

**Affiliations:** Biomedicine Discovery Institute, Monash University, Melbourne, VIC, Australia

**Keywords:** psychoacoustics, auditory perception, psychophysics, environmental sounds, non-linguistic sounds, subjective perception, pleasantness, complexity

## Abstract

Non-linguistic sounds (NLSs) are a core feature of our everyday life and many evoke powerful cognitive and emotional outcomes. The subjective perception of NLSs by humans has occasionally been defined for single percepts, e.g., their pleasantness, whereas many NLSs evoke multiple perceptions. There has also been very limited attempt to determine if NLS perceptions are predicted from objective spectro-temporal features. We therefore examined three human perceptions well-established in previous NLS studies (“Complexity,” “Pleasantness,” and “Familiarity”), and the accuracy of identification, for a large NLS database and related these four measures to objective spectro-temporal NLS features, defined using rigorous mathematical descriptors including stimulus entropic and algorithmic complexity measures, peaks-related measures, fractal dimension estimates, and various spectral measures (mean spectral centroid, power in discrete frequency ranges, harmonicity, spectral flatness, and spectral structure). We mapped the perceptions to the spectro-temporal measures individually and in combinations, using complex multivariate analyses including principal component analyses and agglomerative hierarchical clustering.

## Introduction

The objective features of sensory stimuli form a large part of our subjective perceptions, e.g., a chemical’s structure relates to our perception of its odor (Castro et al., 2013) and the wavelength of light being reflected from an object influences our perception of its color (Solomon and Lennie, 2007). Perceiving these differences in the objective features of stimuli enables us to reliably navigate our worlds, e.g., color perception aids in recognizing the difference between foliage and fruit (Osorio and Vorobyev, 1996). Non-linguistic sounds (NLSs) – e.g., music, a passing bus, snoring, a child crying – are important complex sounds in our everyday environment. Attempts have been made to describe how humans identify and remember such NLSs (e.g., Marcell et al., 2007), and some studies (Kidd and Watson, 2003; Gygi et al., 2004) have used spectrally filtered NLSs to test how qualitative manipulation of the sounds through degraded or limited spectral information affects NLS categorisation, though such manipulations do not directly test precise perceptions of those sounds and do not examine how objective features of the sounds determine perceptions.

Non-linguistic sounds have great advantages for the mapping of perception to objective spectro-temporal features as they are complex, have meaning and are familiar, but do not have the confounding overlay of semantic and linguistic constraints of language. Some studies have probed various perceptual properties of NLSs (Halpern et al., 1986; Ballas, 1993; Penrose and Clark, 1994; Cycowicz and Friedman, 1998; Lewis et al., 2005; Kumar et al., 2008; Reddy et al., 2009; Reuter and Oehler, 2011; Singh, 2011; Kirmse et al., 2012; Talkington et al., 2012) to make findings such as the importance of spectral features to percepts of unpleasantness in NLSs (Halpern et al., 1986; Cox, 2008; Kumar et al., 2008; Reuter and Oehler, 2011). However, almost all of these studies focused on only a single percept and outside of special sets or precepts of sounds such as musical timbre (Grey, 1977; Grey and Gordon, 1978), urgency (Momtahan, 1991; Hellier et al., 1993; Burt et al., 1995; Edworthy et al., 1995; Haas and Edworthy, 1996; Graham, 1999), and identification of materials, e.g., the length of a material being struck and whether it is made of metal or wood (Warren and Verbrugge, 1984; Lakatos et al., 1997), tones (Pollack and Ficks, 1954), or subjects, e.g., the gender of a human walker (Li et al., 1991), little is known generally about the perceptual mappings between complex auditory stimuli and their objective features.

We now address this issue for a database of complex sounds that can have important meaning in everyday life. We calculated objective features of sounds in a large NLSs database and separately asked human participants to record their subjective perceptions for three measures (Complexity, Pleasantness, and Familiarity) which had been used in previous studies of NLS perception (Marcell et al., 2007) but which had not yet been related back to objective features. In addition, we also noted sound identification accuracy. We hypothesized that: (1) the percepts and identification accuracy for NLSs can be quantitatively described by and correlate with objective measurements of specific temporal or spectral contents; and (2) different classes of NLSs would possess unique feature-sets of objective measures which could be related to their perceptual differences or their identification accuracy.

## Materials and Methods

### NLSs Database

Publicly available NLSs (Marcell et al., 2007) and NLSs from online multimedia archive sources (Shafiro and Gygi, 2004) were combined to create a database of 158 sounds. This database contained 144 distinct sound sources with 14 source exemplars (sounds from an identical source, e.g., snoring, but which are distinct recordings or events) from a broad range of categories (Table 1; the full list of sounds used, including their labeled categories, is provided in Supplementary Table 1). The first step was to normalize all sounds to the same amplitude so that amplitude differences, affecting audibility and level, did not affect ratings. It is recognized that this procedure equalizes sounds that, in life, may be of unequal level – e.g., the sound of a car revving up would be naturally louder (unless originating from a great distance) and would be, from our experience of environmental sounds, perceived to be louder than the sounds of birds singing. The Complexity of such relationships as a function of our experience with sounds makes it a very difficult factor to control and that may well be the reason why it has not been accounted for in previous studies (e.g., Gygi et al., 2007; Marcell et al., 2007). Here, it was decided that all sounds would be normalized to a standard RMS amplitude before being used for perceptions. All sounds were normalized to the RMS level of the loudest sound in the database (15 dB) using the Cool Edit 2000 sound program; no other change (e.g., to pitch or rate) was applied.

**TABLE 1:** Categories of sounds in the NLSs database.

Category name and ID number	**Percentage of data set**	**Number of sounds**
Primate (#1)	8.86	14
Animal (#2)	24.05	38
Tool/machine (#3)	17.72	28
Nature (#4)	6.96	11
Human non-voc (#5)	3.80	6
Music (#6)	13.29	21
Insect (#7)	3.16	5
Other (#8)	15.82	25
Explosions/guns (#9)	6.33	10
	*Total*	*158*

*A mathematical approach to correlating objective spectro-temporal features of non-linguistic sounds with their subjective perceptions in humans.*

During analysis of our results, we found it necessary to categorize the NLSs. For categorisation of the NLSs we initially considered using a naïve group of subjects to listen to the NLSs and generate their own categories (c.f. Marcell et al., 2007). However, this methodology still can produce significant heterogeneity: Marcell et al. (2007) report that subjects use widely varying methods, ranging from acoustic similarity to sound imagery or sound source for self-categorization of NLSs, producing widely varying categories even for the same set of NLSs; further, even under the same experimental conditions they can produce many highly specific, small categories. Marcell et al. (2000) also do not show stable and reliable bases for listener-derived groupings of environmental sounds and acknowledge that when listeners are given free classification (unlimited time and unlimited, listener-defined categories) “personal and idiosyncratic categories” (Marcell et al., 2000, p. 852) arise, and even after culling such categories with strict conditions (e.g., categories must be used by >33% of listeners) and manually combining semantically similar groups, e.g., “instrument” and “musical instrument,” 27 categories were left: 4-legged animal, accident, air transportation, bathroom, bird, farm animal, game/recreation, ground transportation, household, human, hygiene, insect, kitchen, machine, musical instrument, nature, paper, pet, reptile/amphibian, sickness, signal, sleep, tool, water/liquid, weapon, weather, and other. Leaving aside the question of how ecologically relevant these distinctions actually are for everyday listeners (e.g., “pet” vs. “4-legged animal” vs. “farm animal”), there is the question of the reliability and stability of the categories (e.g., the sound of a dishwasher could be equally categorized as a “household,” “hygiene,” “kitchen,” “machine,” “water/liquid,” or “other” sound). To determine the latter, Ballas (1993) provided only these 27 categories to a new cohort of listeners to categorize the same sounds. For some sounds, 100% of subjects agreed on the same category but for others, subjects had very little agreement. In fact, only 12 of the 120 sounds reported by Ballas (1993) were categorized with 100% agreement and “[o]verall, 50 sounds were placed with high agreement (90% and above) into categories, 58 were placed with mild-to-moderate levels of agreement (50–89%), and 12 were placed with low levels of agreement (49% and below)” (p. 856). Such high levels of disagreement in a substantial number of categorizations speaks to the use of “personal and idiosyncratic categories” (Marcell et al., 2000, p. 852) and, implicitly, the individual differences which plague listener-derived categorisation of sounds.

Gygi et al. (2007) prescribed listeners to creating at least 5 but not more than 12 categories in total but even with this methodology, one subject’s categorisation was highly specific and had to be discarded. The remaining results were collated and following the method of Marcell et al. (2000) resulted in 13 categories (number indicates number of sounds in that category): animals/people 16, vehicles/mechanical 14, musical 11, water/weather 10, impact/explosion 8, location-specific 6, sports 6, outdoor 4, pitched 3, rhythmic 3, rumbling 3, startling/annoying 2, alerting 2. The number of categories was notably smaller than in Marcell et al. (2000), likely due to the latter using 38 subjects categorizing 120 sounds and the former using 16 subjects categorizing 50 sounds. Gygi et al. (2007) note that in both studies, subjects had a “tendency to categorize sounds based on source types” (p. 851), supporting Gaver’s (1993) hypothesis that everyday listening is primarily oriented to the sound’s source in contrast to “musical listening,” which is primarily orientated toward the sound’s acoustic qualities.

Given that a majority of listeners tend to categorize sounds based on sound source and that there can otherwise be highly specific, small categories and/or disagreement among listeners about which category a sound belongs to, we chose to use a strict experimenter-determined, sound source categorisation method; if the general trend of many sounds leads to many categories is true, then our database of 158 sounds (which is larger than Marcell et al.’s and Gygi et al.’s) could otherwise result in an unwieldy number of categories. We adopted a single categorisation rule, using the source of the NLS as the sole basis for categorisation, and implemented by the two experienced experimenters independently and then consultatively if there were any disagreements. In total, we formed nine categories: primate (*n* = 14 sounds), non-primate animal (*n* = 38 sounds), tool/machine (*n* = 28), non-animal nature (*n* = 11), human non-vocal (*n* = 6), music (*n* = 21), insect (*n* = 5), explosions/guns (*n* = 10), and uncategorized or other (*n* = 25, reflecting that some NLSs did not fit well into any of the other categories). The allocation of our NLSs to these categories is detailed in Supplementary Table 1.

### Psychophysics for Rating of Subjective Perceptions

All psychophysics testing was conducted in a quiet room in an isolated corridor of the department. Ethics approval for the collection of this data was obtained from the institutional Standing Committee on Ethics in Research in Humans.

Twelve normal-hearing observers (7 males, 5 females; mean age 19.8 years, *SD* = 0.57) from the undergraduate student population (all non-musicians), were tested individually using audiometry to ensure normal hearing thresholds across the range from 500 Hz to 8000 Hz (Rajan and Cainer, 2008). Then, in groups of four, participants listened to the 158 NLSs individually, in groups of 20 sounds at a time with a 1-min break between groups, so as not to cause fatigue. Using Windows Media Player the sounds were played out as waveform audio files (.wav) from a Dell Inspiron computer and through an external sound card (Audigy Creative Blaster) to two high-quality speakers (Altec). We used two speakers to model the diotic element used in studies by Gygi et al. (2004), Marcell et al. (2007), and Gygi and Shafiro (2011) but to create a more natural feel than the headphones they used. The four participants were organized in a semi-circle facing the speakers, at a distance of about 1 m from the speakers. After listening to each sound once, the participants were instructed to rate the sound on seven-point Likert scales (Likert, 1932) for “Complexity,” “Pleasantness,” and “Familiarity” (percepts), and were also asked to name the sound source (Accuracy of Naming) by writing down what they thought the sound was and to rate their confidence in identifying the sounds; no verbal interaction between participants was permitted. Subjects were told: “Your task is to identify each sound as quickly and accurately as you can. In one or two words please describe what you hear, and write the appropriate response on the blank sheet. Each sheet has blocks of trial numbers from 1 to 20. Sounds will be presented to you in blocks of 20 and after each block you will receive a 1-min break. After identifying the sound immediately after it is presented, please rate the sound for Familiarity, Complexity, Pleasantness and confidence on a scale of 1 to 7 (e.g., for Pleasantness 1 = not pleasant at all, 7 = highly pleasant). The sound will only be played once so please listen carefully. The time allocated for each sound is 30 s.” This mimicked the method employed by Marcell et al. (2007). Answers were recorded on sheets of paper marked out in blocks of sounds (Block 1 = first 20 sounds), trial number (trial #1, trial #2, etc.), a blank for the name of the sound and three rating scales (Pleasantness, Familiarity, and Complexity). These subjective ratings and sound identification data were then analyzed for correlation with objective measures of the rated NLSs.

### Objective Measures to Define Complex Waveforms

A wide range of temporal and spectral measures of complex waveforms from various disciplines involving signal processing were considered for inclusion in this study. Particular emphasis was placed on those which measured complexity or have been shown to be related to sound percepts relevant or identical to those used in this study. Measures were selected to include a diversity of possible information (including from different information theoretic viewpoints) without being superfluous or repetitive. Since one percept of interest was “Complexity,” we included two entropic measures which measure stimulus “complexity” – sample entropy (Richman and Moorman, 2000; Lake et al., 2002) and permutation entropy (Bandt and Pompe, 2002; Zanin et al., 2012; Riedl et al., 2013), derived from chaos and information theory, respectively. An algorithmic complexity measure was also included, the LZ measure (Xu et al., 1997; Radhakrishnan and Gangadhar, 1998; Zhang et al., 2000; Wu and Xu, 2001; Zhang et al., 2001; Huang et al., 2003; Khalatur et al., 2003; Szczepański et al., 2003; Watanabe et al., 2003). The remaining objective measures have been used in previous research on sound identification or perception: peaks-related measures (Gygi et al., 2007); fractal dimension estimates (Spasić et al., 2005; Shibayama, 2006; Raghavendra and Dutt, 2010), using both the Higuchi method (Higuchi, 1988) and the NLD method (Kalauzi et al., 2009); mean spectral centroid (Grey and Gordon, 1978; Shao et al., 2003; Gygi et al., 2007; Maher and Studniarz, 2012); root mean squares (RMSs) of discrete frequency ranges (Halpern et al., 1986; Gygi et al., 2007; Kumar et al., 2008; Reuter and Oehler, 2011); harmonicity (Yumoto et al., 1982; Boersma, 1993; Lewis et al., 2005; Gygi et al., 2007); spectral flatness (Jayant and Noll, 1984; Boersma, 2001); and spectral structure variability (SSV) or index (SSI) (Reddy et al., 2009; Singh, 2011; Lewis et al., 2012). For a more detailed discussion of these methods, including their derivations, please see Supplementary Material 2.

The HNR measure was calculated for all NLSs using a phonetics research program called Praat (Boersma, 2001) and the remaining 18 selected measures were calculated for all NLSs using MATLAB (MATLAB R2012a, The MathWorks Inc., Natick, MA, United States). MATLAB and Praat codes for calculating these measure may be found at the following GitHub repository: https://github.com/tfburns/sounds-analyses.

### Data Analysis

#### Pair Wise Regression of the Entire Dataset of NLSs

To determine whether any of the perceptions factors or any of the objective measures were interrelated, we conducted linear regressions in pair-wise fashion for all combinations of the four measures: namely, Familiarity, Complexity and Pleasantness, and Accuracy of Naming. Ratings of Familiarity were highly correlated with ratings of Complexity and with Accuracy of Naming. We therefore deleted Familiarity as it did not represent an independent percept, and all further analyses used the perceptions of Complexity and Pleasantness, and the Accuracy of Naming, which we will refer to collectively as our three subjective perceptions – we recognize that, strictly, Accuracy of Naming is not a subjective perception but we include it so to distinguish it from the objective measures of spectro-temporal features against which it was mapped. We examined pair-wise relationships between each subjective measure and each selected objective measure used to define the NLS waveform. Since temporal and spectral domains of sounds appear to be affected differently in different types of hearing loss (Strouse et al., 1998; Probst et al., 2017), and our work may have implications for rehabilitation or testing regimes, we grouped the objective measures into these two domains and conducted pair-wise analyses between each subjective perception and each objective measure within each domain. The resulting regression tables were analyzed to evaluate (see, section “Results” for details) which measures were most independent from the others. Then, separately, we evaluated which objective measures could be chosen as the salient objective measures for later analysis based on their independentness from the other objective measures. We conducted linear regressions for all pair-wise combinations between each salient objective measure versus each of our three subjective perceptions. Significance and correlation coefficients were again calculated for all regressions.

#### Analyses Within Categories of Sound Type

The above analyses on the full NLS dataset identified general trends for the relationships between each salient objective measure and each of our three subjective perceptions. However, there was still a rather large amount of imperfect mapping between the objective measures and each perception. To strengthen these analyses, we examined the homogeneity of the NLSs for their objective measures, and for the three subjective perceptions, to see if stronger relationships might be found by removing outlier NLSs that did not fit a given trend between an objective measure and each of the three subjective perceptions. To objectively and precisely identify these outliers, each NLS was placed into an experimenter-determined sound source category (e.g., Primate sounds, Human non-vocal sounds, Nature sounds, etc. – see Supplementary Table 1 for the full list of categories and segregation of sounds). This allowed us to extract any common objective measures underlying the perception of different types of sounds from within the same sound source category.

One-way ANOVAs were calculated using GraphPad Prism 8 (Graphpad Software) and used to compare the NLS categories for differences in the salient objective measures and differences in their subjective perceptions. Heteroskedasticity was always tested using Brown–Forsythe tests and pair-wise differences between the categories were found using *post hoc* Tukey’s tests. Although the determination of what would be considered ‘homogenous’ would necessarily be somewhat arbitrary, we counted the number of individual significant differences between the categories across all ANOVAs to determine if there were more differences than similarities (where a similarity is defined as having no significant difference between two NLS categories). If there were more differences than similarities, removing or separating categories of NLSs from the database might allow perfect mappings between objective measures and each of our subjective perceptions. If, on the other hand, more similarities than differences were found, mapping improvements would be unlikely.

#### Multi-Variate Analyses, Principal Component Analysis, and Agglomerative Hierarchical Clustering for Data Analyses

In a final analysis, complex, multivariate analyses were used to map combinations of salient objective measures to the three perceptions for NLSs. Multiple linear regressions were first conducted using combinations of objective measures to map onto the perceptions and these relationships visualized with bubble plots and biplots from principal component analysis (PCA). However, as this analysis assumed linear and consistent relationships, separate analyses unconstrained by these assumptions were conducted using agglomerative hierarchical clustering (AHC) with Euclidean distance and Ward’s method, set *a priori*, to divide the database into 10 clusters of NLSs, based on their makeup of objective measures for each domain – spectral and temporal. The 10 clusters were then compared for perceptions, using one-way ANOVAs, to determine if dissimilar combinations of objective measures were related to dissimilar perceptual reports. This analysis was repeated for different degrees of clustering, e.g., clustering all NLSs into five clusters, then three clusters, and finally two clusters (based on their similarities and differences in perceptual ratings, Accuracy of Naming, and salient objective measures). Finally, data were reduced in dimensionality via PCA and then the AHC method employed, to corroborate any significant differences found between clusters in our three subjective perceptions in the high-dimensional cluster analysis described here.

## Results

### Interrelationships Between Familiarity, Complexity and Accuracy and Between Sound Complexity and Accuracy

There were significant inter-relationships between each of the three perceptions and Accuracy of Naming (Table 2; all Pearson correlations *p* < 0.05). Complexity, Familiarity, and Accuracy of Naming had the most robust inter-relationships (*r* > 0.7), while Pleasantness had a weaker relationship with the other percepts and a very weak relationship with Accuracy of Naming. Complexity was inversely correlated with Familiarity: the more complex a sound, the less likely it was to be rated as familiar. Accuracy of Naming was, unsurprisingly, positively correlated with perceptions of Familiarity with the sound, and inversely correlated with perceptions of sound Complexity, a novel relationship that has not, to the best of our knowledge, been previously reported.

**TABLE 2:** Correlation matrix (Pearson) of perceptions and identification accuracy.

**Variables**	**Complexity**	**Pleasantness**	**Accuracy**	**Familiarity**
Complexity		−**0.283**	−**0.701**	−**0.807**
Pleasantness	−**0.283**		0.197	**0.321**
Accuracy	−**0.701**	0.197		**0.735**
Familiarity	−**0.807**	**0.321**	**0.735**	

^*^All pearson correlations had *p* < 0.05 and bolded values indicate highly significant relationships (α = 0.001).

Given that Familiarity was so highly correlated with Accuracy of Naming and Complexity, it provided the least amount of independent variation and so was excluded from subsequent analyses. Although Accuracy of Naming appeared to depend on Complexity, it was retained in further analysis, given the novelty and non-intuitive nature of this relationship (i.e., why Accuracy of Naming should be inversely correlated with Complexity). Overall, we retained for subsequent analyses three measures: the two percepts of Complexity and Pleasantness, and the outcome measure of Accuracy of Naming.

### Perceptual Relationships With Individual Objective Measures

To characterize the NLSs objectively, we started with 19 objective measures that have been used to characterize complex signals. We calculated these measures for our NLSs and conducted pair-wise linear regression analyses of one measure against another, for all combinations. As expected, many objective measures which were theoretically related or which measured similar qualities of an NLS were significantly correlated with one another for our NLS database too, e.g., HNR and SFM (*r* = −0.822); Higuchi and NLD estimates (*r* = 0.812). Hence, to avoid redundancy of information captured by the objective measures and to increase the power of subsequent analyses, we identified a set of salient objective measures which (for our dataset) accounted for large proportions of the variance in other objective measures within the same domain (i.e., spectral or temporal) – i.e., we first identified the objective measures that correlated most highly with each other.

Although there are many significant relationships among the measures, some are quite weak (e.g., Higuchi FD estimate and duration, *r* = 0.059) and the choice of measures should include some consideration toward the strength of relationships. Any such consideration would have to be made on an arbitrary basis since the “strength” of a correlation is subjective and relative; we decided that if the correlation between two measures was ≥±0.45, only one of the pair would be included in subsequent analyses. Ten cross-relationships with *r* ≥ ± 0.45 were found among the temporal measures and seven among the spectral measures. Retaining only one from each such cross-relationship reduced the 19 objective measures to a subset of seven salient objective measures (four temporal and three spectral; listed in Table 3) which, independent of each other, accounted for a large proportion of the total variance among all of the measures.

**TABLE 3:** Correlation matrix (Pearson) for Complexity, Pleasantness, and Accuracy of Naming, showing their individual, pair-wise relationships with each of the salient measures.

Salient measures	Temporal/Spectral	**Accuracy**	**Complexity**	**Pleasantness**
NLDFD	Temporal	−**0.254**	**0.265**	−0.057
Mean peak	Temporal	−0.038	−0.039	**0.324**
Permutation entropy	Temporal	−0.031	0.106	−0.034
LZ complexity (differential binary)	Temporal	−0.11	**0.162**	−0.048
HNR	Spectral	**0.182**	−**0.248**	0.109
Mean spectral centroid	Spectral	−**0.35**	**0.293**	−0.089
1000–2000 Hz RMS	Spectral	0.076	−0.052	−0.021

^*^Bolded values indicate significant relationships (at α = 0.05).

These salient objective measures were used to correlate with the three measures (Complexity, Pleasantness, and Accuracy of Naming) of the NLSs. The outcome of these correlations is shown in Table 3. The mean spectral centroid was the best individual descriptor for both Accuracy of Naming (*p* < 0.0001) and Complexity (*p* = 0.0002). The only salient measure to individually correlate significantly with Pleasantness was the temporal measure of mean peak relative amplitude (*p* < 0.0001), which also did not significantly correlate with the Accuracy of Naming or Complexity.

Two measures – one temporal (permutation entropy) and one spectral (1000–2000 Hz RMS) – did not correlate significantly with any of the three measures. Note also that whenever a measure was significantly correlated with Accuracy of Naming, it was also significantly correlated with Complexity, except in the case of the temporal measure of LZ complexity (differential binary), which was only significantly correlated with Complexity (*p* = 0.0413).

In summary, there was no strong correlation between any single salient objective measure and the perceptions of Complexity and Pleasantness of NLSs or the Accuracy of Naming the NLSs.

### Analysis of NLSs Within Categories: Reducing Database Homogeneity

The absence of strong correlations between any single salient objective measure and our three subjective measures could be due to heterogeneity in the NLSs database. The NLSs span a wide variety ranging from machine noises to primate and animal vocalizations, and significant heterogeneity in the overall database may warrant analyzing NLSs in smaller, alike groups. It was for this reason that we categorized the NLSs as described in Materials and Methods.

One-way ANOVAs with *post hoc* Tukey’s tests revealed significant (*p* < 0.05) differences between the NLS categories for our subjective measures and salient objective measures. Significant differences between the NLS categories were found for Accuracy of Naming (1 significant difference), Complexity (4 significant differences), and Pleasantness (12 significant differences). For the salient objective measures, differences between the NLS categories were found for permutation entropy (1 significant difference), the differential binary coded LZ complexity (3 significant differences), and HNR (11 significant differences), but not for NLD fractal dimension, mean peak amplitude, 1000–2000 Hz RMS, or mean spectral centroid. The most prominent differences among the sounds were therefore in the domains of Pleasantness and HNR.

These significantly different pairs represented only one quarter of the total possible pair-wise differences among the NLS categories for individual percepts or objective measures, i.e., the NLS categories had fewer significant differences than there was potential for (by a factor of three) in perceptual ratings, Accuracy of Naming, and the salient objective measures. Further, the differences between NLS categories for perceptual ratings and Accuracy of Naming were inconsistent between the same NLS categories for salient objective measures.

These analyses do not support the hypothesis that the NLSs database should be treated as categories of sounds, based on sound source. Hence, it will be assumed that the NLSs can be treated as a single group of sounds which have an acceptable homogeneity. It is recognized that there were some perceptual and objective differences between the categories vis-à-vis Pleasantness and HNR and for this reason, in relevant subsequent analyses, information on labeled sound categories are provided.

### Multivariate Analysis

Given that pair-wise regressions of even the reduced subset of most salient objective measures, against any of the three subjective measures, did not yield strong relationships, we examined if combinations of objective measures would yield better predictions.

#### Pairs of Measures

**FIGURE 1:**
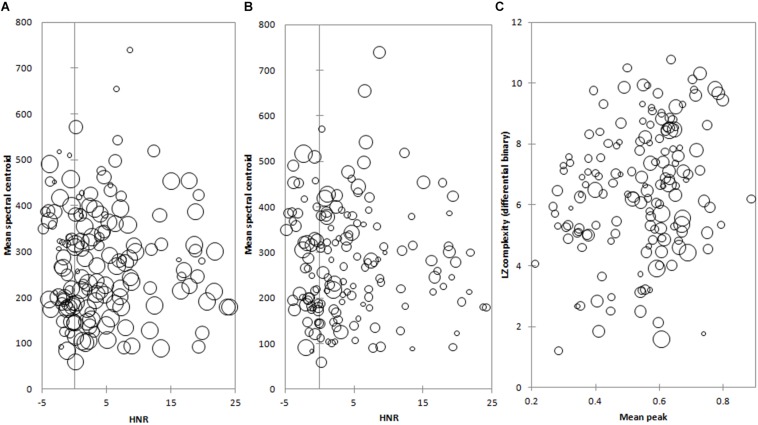
**(A)** Mean spectral centroid and HNR measures bubble plot, where the size of a bubble (representing an individual NLS) is scaled to its accuracy of naming. **(B)** Mean spectral centroid and HNR measures bubble plot, where the size of a bubble (representing an individual NLS) is scaled to its complexity. **(C)** Mean peak and LZ complexity (differential binary) measures bubble plot where the size of a bubble (representing an individual NLS) is scaled to its pleasantness.

Multiple linear regression using pairs of measures to explain each of the subjective measures of Complexity and Pleasantness and the Accuracy of Naming the NLSs, provided more significant relationships (c.f. Table 3 vs. Table 4). In keeping with the distinction made previously between temporal and spectral domains, we only combined salient measures from the same domain (spectral or temporal). These analyses are summarized in Table 4 and plotted as bubble plots in Figure 1 for the regressions with the highest explanatory power for each percept and Accuracy of Naming.

**TABLE 4:** Correlation matrix (*R* ^2^) for Complexity, Pleasantness, and Accuracy of Naming, showing relationships with pairs of salient measures.

Pairs of salient measures	**Accuracy**	**Complexity**	**Pleasantness**
NLDFD + permutation entropy	**0.064**	**0.076**	0.004
NLDFD + mean peak	**0.065**	**0.074**	**0.112**
NLDFD + LZ complexity (differential binary)	**0.071**	**0.087**	0.005
Permutation entropy + mean peak	0.002	0.014	**0.111**
Permutation entropy + LZ complexity (differential binary)	0.012	0.030	0.003
Mean peak + LZ complexity (differential binary)	0.012	0.033	**0.121**
HNR + mean spectral centroid	**0.152**	**0.144**	0.019
HNR + 1000–2000 Hz RMS	0.036	**0.062**	0.013
Mean spectral centroid + 1000–2000 Hz RMS	**0.130**	**0.089**	0.008

^*^Bolded values indicate significant relationships (α = 0.05).

The spectral combination of HNR and mean spectral centroid provided the largest explanation of both Accuracy of Naming (Figure 1A; *R* ^2^ = 0.152; *p* < 0.0001) and Complexity (Figure 1B; *R* ^2^ = 0.144; *p* < 0.0001) of the pair-wise combinations. NLSs which were less frequently identified correctly also tended to have a lower HNR value (Figure 1A); equally, NLSs with lower HNRs were more frequently rated as having high Complexity (Figure 1B). Thus, low HNRs appear to be a consistent factor accounting for the above-noted inverse inter-relationship between Complexity and Accuracy of Naming, the latter being a novel relationship that not previously reported.

Mean spectral centroid also appeared to separate NLSs broadly – more complex and less well identified NLSs tended to have higher mean spectral centroids (Figure 1B).

The temporal combination of mean peak relative amplitude and LZ complexity (differential binary) had the most explanatory power for the perception of Pleasantness of NLSs (Figure 1C; *R* ^2^ = 0.121; *p* < 0.0001). NLSs with a high Pleasantness where situated mainly on the higher end of the mean peak relative amplitude axis. However, there was no clear distinction for bubble size along the LZ complexity (differential binary) axis, where there seemed to be a fairly equal representation of pleasant and unpleasant NLSs.

#### Three or More Measures

Adding additional objective measures into the multiple linear regressions only marginally increased the predictive power for each domain of the three perceptions (Table 5 c.f. Table 4), suggesting that assuming linearity in the analysis reached the limit of our predictive capabilities using just the salient measures.

**TABLE 5:** Correlation matrix (*R* ^2^) for Complexity, Pleasantness and identification accuracy, showing their relationships with groups of salient measures.

Salient measures grouped by domain	**Accuracy**	**Complexity**	**Pleasantness**
All salient temporal measures – NLDFD + permutation entropy + mean peak + LZ complexity (differential binary)	**0.071**	**0.098**	**0.128**
All salient spectral measures – HNR + mean spectral centroid + 1000–2000 Hz RMS	**0.157**	**0.145**	0.020

^*^Bolded values indicate significant relationships (α = 0.05).

**FIGURE 2:**
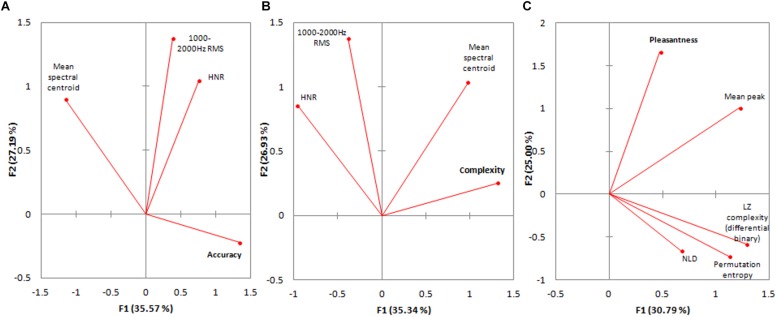
**(A)** Biplot of PCA for the salient spectral measures which best described accuracy of naming. N.B. Only 62.76% of the variance within these variables is represented. **(B)** Biplot of PCA for the salient spectral measures which best described complexity. N.B. Only 62.27% of the variance within these variables is represented. **(C)** Biplot of PCA for the salient spectral measures which best described pleasantness. N.B. Only 55.79% of the variance within these variables is represented.

The relationships between the measures which best accounted for each of the three subjective measures were visualized using PCA biplots (Figure 2). With regards to Accuracy of Naming of the NLSs (Figure 2A), HNR and 1000–2000 Hz RMS are relatively orthogonal (unaligned) to Accuracy of Naming and the mean spectral centroid lies on the same plane but is almost exactly opposite in direction. With respect to the percept of Complexity of the NLSs, Figure 2B shows that both the 1000–2000 Hz RMS measure and HNR are nearly orthogonal to Complexity, and the mean spectral centroid is more closely aligned along the plane of Complexity. Finally, with respect to the percept of Pleasantness of the NLSs, Figure 2C shows that mean peak relative amplitude is the primary measure to align itself roughly with Pleasantness and all of the other salient temporal measures are relatively unrelated, or contribute weakly.

#### Agglomerative Hierarchical Clustering (AHC)

The resultant clusters of the AHC on the raw spectral salient objective measures data for all sounds, differed significantly (one-way ANOVA and *post hoc* Tukey’s test; *p* < 0.05). However, the objective spectral properties of the NLSs defined NLS groupings for Accuracy of Naming and perceptual Complexity, but not for perceptual Pleasantness. More precisely, these differences were found for Accuracy of Naming (two: *p* = 0.0441; *p* = 0.0441) at the 10-cluster level, for Accuracy of Naming (two: *p* = 0.0014; *p* = 0.0016) and Complexity (two: *p* = 0.0039; *p* = 0.0069) at the five-cluster level, and for Accuracy of Naming (one: *p* = 0.0001) and Complexity (one: *p* = 0.0008) at the three-cluster level. However, the difference at the 10-cluster level for Accuracy of Naming relied on one cluster having only two NLSs. Importantly, no significant differences were found between clusters for Pleasantness at any level of clustering. This is consistent with our previous analyses that spectral measures are strongly related to both Accuracy of Naming and Complexity, but not to Pleasantness (Tables 3–5).

The resultant clusters from the raw temporal AHC showed no significant differences at the 10- or four-cluster level but at the two-cluster level showed significant differences for both Accuracy of Naming and Complexity. These differences had, however, much higher *p* -values (*p* = 0.0306 for Accuracy of Naming and *p* = 0.0470 for Complexity) than those from a comparable cluster level for the spectral AHC, and were similar to the three-cluster level (where *p* = 0.0001 for Accuracy of Naming and *p* = 0.0008 for Complexity). This is consistent with our earlier results that temporal measures do not correlate as strongly as spectral measures with Accuracy of Naming and Complexity (Tables 3–5).

Principal component analysis were conducted using all temporal measures and, separately, all spectral measures. The first two principal components (PCs), which accounted for the greatest proportions of variance within the original sets of salient measures, were then selected for use in subsequent (PC) AHC analyses. In the case of the spectral PCA, the first two PCs accounted for 70.78% of the variance, and for the temporal PCA, 61.37% of the original variance was represented in the first two PCs.

Results for the AHC analysis using PCs derived from the spectral PCA were similar to those from the raw spectral AHC (which used the unmodified data) – there were differences at multiple cluster levels for Accuracy of Naming and Complexity, but not for Pleasantness. The differences were found for Accuracy of Naming (two: *p* = 0.0296; *p* = 0.0167) and Complexity (two: *p* = 0.0218; *p* = 0.0258) at the 10-cluster level, for Accuracy of Naming (two: *p* = 0.0008; *p* = 0.0176) and Complexity (two: *p* = 0.0008; *p* = 0.0176) at the four-cluster level, but none at the two-cluster level. However, at both 10- and four-cluster levels, the clusters in the ANOVAs for Accuracy of Naming and Complexity had significantly different standard deviations (*p* < 0.05). Thus, these results may be unreliable but are presented in the interest of completeness.

Results for the AHC analysis using PCs derived from the temporal PCA were similar to those from the previous raw temporal AHC – there were differences at multiple cluster levels for Accuracy of Naming and Complexity, but never for Pleasantness. The differences were for Accuracy of Naming (one: *p* = 0.0491) at the 10-cluster level, for Accuracy of Naming (one: *p* = 0.0296) at the six-cluster level, and for Accuracy of Naming (one: *p* = 0.0027) and Complexity (one: *p* = 0.0115) at the three-cluster level. That absence of significant differences in Pleasantness in this analysis (as was expected) is possibly due to the fact that only 61.37% of the variance was captured in the temporal PCA. Of our salient measures, mean peak relative amplitude is the major or sole contributor to the relationship with Pleasantness and the objective temporal domain. It could be that its contribution was “washed-out” by the lost overall variance or variance contribution from the other salient temporal measures included in the PCA conducted prior to the AHC.

## Discussion

To our knowledge this study is the first major attempt at understanding the way humans perceive the complexity of NLSs in relation to the objective features of those sounds. Two near-orthogonal axes were identified in perceptual space and we have added significantly to what is known about the objective determinants of the percepts of Pleasantness, Familiarity, and NLS identification. We also demonstrated the usefulness of AHC analysis on transformed data such as these and how similar methods such as artificial neural networks may help to further tease out complex mappings between objective features of stimuli and their subjective perception as reported by humans.

### Relationships Among Subjective Percepts and Accuracy of Naming

As noted earlier, other studies have probed various perceptual properties of NLSs (Halpern et al., 1986; Ballas, 1993; Cycowicz and Friedman, 1998; Lewis et al., 2005, 2012; Marcell et al., 2007; Kumar et al., 2008; Reddy et al., 2009; Reuter and Oehler, 2011; Singh, 2011; Kirmse et al., 2012) but most focused on only a single percept. Ballas (1993) used the most exhaustive list of perceptual properties, of 22 ratings scales, for 41 NLSs, and condensed this battery of perceptions into three PCs representing 87% of the variance. Given the number of different rating scales, this result shows that subjective ratings can be highly interrelated or interdependent. These ratings did not include Accuracy of Naming, nor Complexity or Pleasantness of NLSs, all of which we considered here. Ballas (1993) did consider Familiarity but the methodology allowed participants to replay the sound as many times as desired and this could affect the other perceptual reports – e.g., Familiarity with an NLS can alter the way it is processed (Cycowicz and Friedman, 1998; Kirmse et al., 2012). Marcell et al. (2007) studied the perceptions of Complexity, Pleasantness, and Familiarity, and the Accuracy of Naming but did not attempt to correlate these perceptual ratings.

We attempted to correlate the percepts of Complexity, Pleasantness, Familiarity, and the Accuracy of Naming. For our dataset of NLSs, there was a dominant relationship between the percepts of Complexity and Familiarity, and Accuracy of Naming: sounds rated as being highly complex are difficult to accurately name and are rated as not familiar, and vice-versa. With the constraint that our subjects were all from a very similar Western industrialized background (albeit of different ethnicities), this indicates that a person’s auditory experience determines their ability to identify NLSs, and “complex” NLSs are rated as such due to a person’s lack of Familiarity with them, independent of any objective characteristics of the sound.

Pleasantness was not well related with the other percepts and especially not with the Accuracy of Naming, except for a weak relationship whereby familiar sounds were rated as slightly more pleasant. Halpern et al. (1986) suggested that the functional purpose of unpleasantness as an auditory feature is to communicate distress or warnings, but this hypothesis was based on similarities between the properties of unpleasant sounds and macaque monkey warning cries, and these similarities are not sufficiently robust to support this hypothesis (Cox, 2008). McDermott and Hauser (2004) found that cotton-top tamarins (*Saguinus oedipus* ) had no preference for amplitude-matched white noise versus the sound of a three-pronged metal garden tool scraped down a pane of glass (a “screech” sound comparable to fingernails scraping down a blackboard; Halpern et al., 1986) while humans overwhelmingly preferred the white-noise control (McDermott and Hauser, 2004) despite both species sharing many similarities in perceptual processing for vocalizations (Ramus et al., 2000; Miller et al., 2001a, b; Newport et al., 2004). McDermott and Hauser (2004) also showed that the tamarins significantly preferred looping soundscapes composed of tamarin food chirp sounds versus tamarin distress screams. If the perception of unpleasantness is species-specific, it might not be possible to define it in terms of objective properties alone (see below for discussion of the objective properties related to Pleasantness in humans) or may involve semantic or emotive contents which cannot be fully interpreted by another species, or both.

Highly pleasant sounds, such as nature sounds (Shimai, 1993; Marcell et al., 2007; Kumar and Ohana, 2008; Alvarsson et al., 2010), may even impact on health (Cohen et al., 2007). Alvarsson et al. (2010) show that sounds such as nature sounds (consistently rated as highly pleasant; Shimai, 1993; Marcell et al., 2007; Kumar et al., 2008; Alvarsson et al., 2010), facilitate recovery from sympathetic activation in humans after experiencing a psychological stressor but that other sounds, such as road traffic sounds (often rated as unpleasant; Marcell et al., 2007; Alvarsson et al., 2010), do not facilitate the same recovery. This facilitation or absence of recovery may (also) involve higher-order emotional or subjective elements and not just be due to the objective features of these NLSs, especially considering that cortical representation of unpleasant sounds is influenced by interactions with the amygdala (Kumar et al., 2012).

### Relationships Among Objective Measures of NLSs

Many correlations were found among the objective measures in both temporal and spectral domains, some expected and some not. These relationships may only be true for this dataset of NLSs and not a general rule for all types of NLSs, let alone all types of signals, although some of these relationships are also evident in EEG signals (Burns and Rajan, 2015).

Although dissimilar in methodology, both fractal dimension estimation techniques – Higuchi and NLD – were highly correlated, as also seen when these analyses were originally applied to EEG waveforms (Kalauzi et al., 2009). Interestingly, we have reported the first significant correlation between a fractal dimension estimate and the LZ complexity measure (average binary and modified zone binary). Since both measures ultimately attempt to find self-similarity or repeating aspects in a signal, it is not unexpected that they carry some common information. However, the degree of common information was not always similar, e.g., the Higuchi FD correlated poorly with LZ complexity (modified zone binary) (*r* = 0.361; Table 3) whereas the NLD FD was more correlated (*r* = 0.577; Table 3).

The most significant relationship among the spectral measures, the negative relationship between the SFM and HNR, is logical since if a sound is highly harmonic it cannot also be “flat” in its spectrum. The fact that the SFM and SSI are related explains why they were also well correlated and therefore why there was also a relationship between SSI and HNR.

### Categories of Sounds Differentiated by Source Were Not Strongly Differentiated by Either Subjective Percepts or Objective Measures

We analyzed our NLSs database for homogeneity to examine if stronger relationships could be found by removing outlier NLSs which did not fit a given trend between an objective measure and subjective percept or Accuracy of Naming. We sought to determine if different categories of NLSs followed their own trends, independently of other categories (see section “Analyses Within Categories of Sound Type”).

With respect to Accuracy of Naming, non-linguistic human vocalizations (which we classed under “primates”) were the most accurately identified NLS category, although only the “other” category was significantly less so (*p* = 0.0037 c.f. “primates”). This differs slightly from the results of Inverso and Limb (2010), who found “mechanical/alerting” sounds to be slightly more recognizable than “human” sounds. However, their “human” category also included non-vocalizations like the sound “footsteps” and their study participants were experienced cochlear implant (CI) users, not normal-hearing university students. It is not known if the perception of NLSs is the same between the two categories of people, but these findings raise caveats about assuming that the understanding of the perception of NLSs by normal-hearing subjects can directly translate to the perception of NLSs by deaf subjects using a CI.

With respect to perceived complexity, primate sounds in our database were rated as significantly less complex than the sounds of other animals; this may reflect the similarity between human sounds and primate sounds (Hauser and Tecumseh Fitch, 2003), and therefore be a Familiarity factor.

For ratings of Pleasantness, for our NLS categories, nature and music sounds were rated as the most pleasant, as in previous studies (Shimai, 1993; Marcell et al., 2007; Kumar et al., 2008; Alvarsson et al., 2010).

Finally, the only salient objective measures showing differences among the different NLS source categories were LZ complexity (differential binary) and the HNR. Previous studies (Gygi et al., 2007; Lewis et al., 2012; Leaver and Rauschecker, 2010) have noted the importance of harmonicity to NLS classification but did not determine if there were differences among different experimenter-determined categories of sounds for HNR (or LZ complexity).

Overall, there were some significant differences among different categories of NLS sources for subjective percepts and for objective measures of sounds but there were many groups for which no significant differences were observed for either. Indeed, we found that objective features of sounds can be similar or even identical between different source categories. Later categorization of the different sounds using AHC analyses showed that this method of objective categorizing was more revealing of similarities and differences between different sounds (note: not different sound categories) for Complexity and the Accuracy of Naming. Thus, identifying a sound’s source appears only partly to rely on objective measures, and other information, perhaps visual integrative learning or memory (Thompson and Paivio, 1994; Giard and Peronnet, 1999), may also play a role. Higher-order associations may also provide context and input into perceptions of Complexity and Pleasantness (Johnson et al., 1999).

This conclusion has implications for the identification of NLSs by CI users (viz., Inverso and Limb, 2010; Shafiro et al., 2015). The poor success of CI users in identifying NLSs may have less to do with the objective properties of the NLSs and more to do with other attributes, e.g., remembering and associating that sound with an image, based on experience or emotional valency. Work on the perceptual learning of spectrally degraded speech and NLSs (Loebach and Pisoni, 2008) shows that speech training does not generalize to NLSs but that NLS training does generalize to speech. This suggests that the development of specific NLS training programs for CI or hearing aid users would be greatly beneficial, both to allow recognition of an important set of everyday sounds with strong emotional or survival value and to feed into speech recognition.

### Complexity, Familiarity, and Accuracy of Naming

Since different categories of sounds, segregated by sound source, were not strongly differentiated by either subjective percepts or objective measures, we used the entire NLSs database to find correlations with the objective measures along our two, near-orthogonal perceptual dimensions of Complexity and Pleasantness. Since Familiarity closely matched Complexity and the Accuracy of Naming, any relationship which was true for one would often exist oppositely for the other. (Pleasantness was a near-orthogonal perceptual dimension to the Complexity-Familiarity-Accuracy-of-Naming dimension, and its relationships with objective measures are discussed in the section “Pleasantness.”)

The consistent correlation between objective measures and Familiarity suggests either (1) our subjects controlled their auditory experience with respect to objective features of NLSs (e.g., they avoided sounds with high FDs, and thus were unfamiliar with them) or (2) there is something intrinsic to these objective features which makes NLSs easier to become familiar with. The latter hypothesis seems more plausible.

Our standard multivariate analyses showed that both temporal and spectral measures can be associated significantly with differences in Complexity and the Accuracy of Naming (Tables 4, 5). When the sounds in our database were separated based on these objective measures using AHC analyses (and incorporating PCA), differences arose between the resultant clusters for both Complexity and the Accuracy of Naming. However, the resultant clusters from AHC analyses which included only spectral measures tended to show greater numbers of and more significant differences in Complexity and Accuracy of Naming. This suggests humans may assess spectral features, more than temporal features, when assessing NLS Complexity, Familiarity, or when seeking identify it. Favoring spectral features over temporal ones may be a more efficient means of neural processing insofar as being able to more readily make a subjective judgment about a NLS, since relying on temporal features may require a longer time exposure to the NLS.

### Pleasantness

For ratings of Pleasantness, temporal measures or combinations thereof had the highest explanatory power and none of the salient spectral measures were significantly correlated – alone or in combination – with Pleasantness; clusters created using spectral measures did not show any significant differences in Pleasantness. The fact that temporal information becomes highly important in CI users with spectrally degraded stimuli (Fu et al., 2004), and because a decreased level of music appreciation is found in the same individuals (Nimmons et al., 2008; Philips et al., 2012), makes it likely that temporal features are important to the appreciation of sounds as being pleasant. Poorer temporal resolution might mean that such features are indiscernible to a person with hearing loss (Francart et al., 2015), and thus result in a lowered appreciation of music.

However, past studies have repeatedly shown the importance of spectral features to percepts of Unpleasantness in NLSs (Halpern et al., 1986; Cox, 2008; Kumar et al., 2008; Reuter and Oehler, 2011). This might be because Unpleasantness has different objective drivers than does Pleasantness, i.e., the presence of an objective feature might make something unpleasant but its absence might have a neutral or null effect on Pleasantness. However, it must also be recognized that there are significant methodological differences between our study and the previous studies: Halpern et al. (1986) and Reuter and Oehler (2011) used 16 digitally resynthesized and filtered NLSs; Kumar et al. (2008) used 75 auditory representations of NLSs in a modeled primary auditory cortex (Shamma, 2003); and Cox (2008) used 34 NLSs but did not measure any objective features. Further, none of these studies tested the main temporal measure which we found to correlate with Pleasantness – mean peak relative amplitude. Based on the latter difference we would argue that these studies do not negate our finding that Pleasantness perception is based on temporal measures. This is not to say that it has no spectral bases, but rather that it at least has some temporal ones.

## Conclusion

This study represents a critical step to the principled subjective and objective characterization of NLSs, and could allow NLSs libraries to be used in the evaluation or training of people with hearing or cognitive impairments in the processing of NLSs or their features.

As shown here and in other perceptual studies, e.g., for color perception (Rúttiger et al., 1999) and auditory pitch (Tramo et al., 2005), objective features of sensory data do not always map simply to human perception – they can have complex mappings and interactions. To fully unravel such relationships, methods like the use of artificial neural networks have been employed with some success (Shao et al., 2003). Such analytical complexity is despite the fact that these subjective perceptions are otherwise simple for us to understand and report, e.g., “the apple is red, not blue,” “that C# is from a violin, not a piano.” While we have shown that a variety of objective measures and percepts (including the Accuracy of Naming) have strong interrelationships for our NLSs database, future studies may still wish to investigate an even broader range of percepts, objective features, and their interactions for the same or an expanded database.

One practical implication of a NLSs database is in the creation of a NLSs hearing test or training regime. Information found by a NLSs hearing test could identify if a person finds temporal or spectral information more difficult to process, and this (married with a NLS training regiment) could aid clinicians in developing more specific treatments and rehabilitation strategies for their patients. Another important area is the way in which perceptions may change with cognitive impairment such as in dementia or less advanced diseases. Whether the perception of the NLSs we used is the same under such conditions and, if not, what elements of our mathematical descriptors would best fit the altered perception is an open question. Rather than speculate on this complex topic, we hope to address this question directly in future studies.

Another potential benefit could be in the identification and removal of NLSs where they represent unwanted background noise, such as in imperfect binary algorithms to digitally separate speech from noise. Such algorithms could be incorporated into hearing aids or CIs to dramatically counteract one of the most commonly complained symptoms of hearing loss (the cocktail party effect).

## Data Availability

The raw data supporting the conclusions of this manuscript will be made available by the authors, without undue reservation, to any qualified researcher.

## Ethics Statement

This study was carried out in accordance with the recommendations of the Monash University Standing Committee on Ethics in Research in Humans with written informed consent from all subjects. All subjects gave written informed consent in accordance with the Declaration of Helsinki. The protocol was approved by the Monash University Standing Committee on Ethics in Research in Humans.

## Author Contributions

TB analyzed the data and wrote the manuscript. RR designed and performed the research, analyzed the data, and wrote the manuscript.

## Conflict of Interest Statement

The authors declare that the research was conducted in the absence of any commercial or financial relationships that could be construed as a potential conflict of interest.

## Abbreviations

AHC: agglomerative hierarchical clustering
CI: cochlear implant
EEG: electroencephalogram
FD: fractal dimension
HNR: harmonics-to-noise ratio
LZ: Lempel-Ziv
NLD: normalized length density
NLSs: non-linguistic sounds
PC: principal component
PCA: principal components analysis
RMS: root mean square
SSI: spectral structure index
SSV: spectral structure variability.
