# Supplementary material for: A Mathematical Approach to Correlating Objective Spectro-Temporal Features of Non-linguistic Sounds With Their Subjective Perceptions in Humans
Source: Front Neurosci. 2019 Jul 31;13:794. doi: 10.3389/fnins.2019.00794 (PMC6685481; doi:10.3389/fnins.2019.00794)
Supplement: Supplementary file 1 [file Table_1.DOCX]

SUPPLEMENTARY MATERIAL

| Supplementary Table 1. List of sounds used and their labelled categories.  (Sounds from Marcell et al., 2007 and additional online multimedia archive sources) |  | |
| --- | --- | --- |
| *File name* | *Category* | |
| Airplane |  | #3 |
| Baby cry |  | #1 |
| Barn yard |  | #2 |
| Basketball |  | #8 |
| Bee |  | #7 |
| Bee swarm |  | #7 |
| Bike start |  | #3 |
| Bird chirp |  | #2 |
| Birds chirping |  | #2 |
| Blowing nose |  | #5 |
| Boeing 707 |  | #3 |
| Bowling |  | #8 |
| Brushing teeth |  | #8 |
| Burnout |  | #3 |
| Burp |  | #5 |
| Camera |  | #3 |
| Can crush |  | #8 |
| Car crash |  | #9 |
| Car horn |  | #6 |
| Cash register |  | #3 |
| Cat |  | #2 |
| Cat meow |  | #2 |
| catpurr2 |  | #2 |
| Cat scream |  | #2 |
| Cat yell |  | #2 |
| Chewing |  | #5 |
| Chickens |  | #2 |
| Child cough |  | #1 |
| Chimpanzee |  | #1 |
| Church bell |  | #6 |
| Clapping |  | #5 |
| Clear throat |  | #1 |
| Coin drop 1 |  | #8 |
| Coin drop 2 |  | #8 |
| Cow |  | #2 |
| Crank engine |  | #3 |
| Crickets |  | #7 |
| Crow |  | #2 |
| Crow |  | #2 |
| Crunch |  | #8 |
| Cuckoo Clock |  | #2 |
| Cymbals |  | #6 |
| Dishes break |  | #8 |
| Dog bark |  | #2 |
| Dog growl |  | #2 |
| Dolphin |  | #2 |
| Donkey |  | #2 |
| Doorbell |  | #6 |
| Door close |  | #8 |
| Drop ice |  | #8 |
| Drums |  | #6 |
| Duck |  | #2 |
| Elephant |  | #2 |
| Explosion 1 |  | #9 |
| Explosion 2 |  | #9 |
| Fart |  | #5 |
| Fire crackers |  | #9 |
| Fire cracker |  | #9 |
| Fire truck |  | #3 |
| Flute |  | #6 |
| Frog croak 1 |  | #2 |
| Frog croak 2 |  | #2 |
| Frying food |  | #8 |
| Gargling |  | #5 |
| Glass breaking |  | #8 |
| Gong |  | #6 |
| Guitar |  | #6 |
| Gunshots |  | #9 |
| Hairdryer |  | #3 |
| Hammering |  | #3 |
| Harmonica |  | #6 |
| Harp |  | #6 |
| Hawk |  | #2 |
| Helicopter |  | #3 |
| Horn |  | #6 |
| Horse gallop 1 |  | #8 |
| Horse gallop 2 |  | #8 |
| Horse neigh |  | #2 |
| Honda engine |  | #3 |
| Hurricane |  | #4 |
| Hurt dog |  | #2 |
| Jackhammer |  | #3 |
| Jet takeoff |  | #3 |
| Jungle birds |  | #2 |
| Kiss |  | #8 |
| Knocking |  | #8 |
| Laughing 1 |  | #1 |
| Laughing 2 |  | #1 |
| Lawnmower |  | #3 |
| Lion |  | #2 |
| Locust |  | #7 |
| Machinegun 1 |  | #9 |
| Machinegun 2 |  | #9 |
| Mazda horn |  | #3 |
| Mice |  | #2 |
| Monkey 1 |  | #1 |
| Monkey 2 |  | #1 |
| Mosquito |  | #7 |
| Motorcycle |  | #3 |
| Ocean |  | #4 |
| Organ |  | #6 |
| Owl |  | #2 |
| Panther |  | #2 |
| Pencil sharpener |  | #3 |
| Piano |  | #6 |
| Pig |  | #2 |
| Pinball |  | #3 |
| Ping pong |  | #8 |
| Pouring water |  | #4 |
| Rain |  | #4 |
| Rain hitting roof |  | #4 |
| Rattle snake |  | #2 |
| Rooster |  | #2 |
| Running water |  | #4 |
| Sawing |  | #3 |
| Sax |  | #6 |
| Scream |  | #1 |
| Seagull |  | #2 |
| Seal |  | #2 |
| Sheep |  | #2 |
| Shotgun |  | #9 |
| Shuffling cards |  | #8 |
| Sky rocket |  | #9 |
| Sneeze |  | #1 |
| Snoring 1 |  | #1 |
| Snoring 2 |  | #1 |
| Sonar beep |  | #6 |
| Sparrow |  | #2 |
| Stapler |  | #3 |
| Swords |  | #8 |
| Tea kettle |  | #8 |
| Tearing paper |  | #8 |
| Telephone ring |  | #6 |
| Thunder |  | #4 |
| Tire spin |  | #3 |
| Toilet |  | #8 |
| Train |  | #3 |
| Truck starting |  | #3 |
| Truck engine |  | #3 |
| Trumpet |  | #6 |
| Turkey |  | #2 |
| Turn page |  | #8 |
| Typewriter |  | #3 |
| Velcro |  | #8 |
| Violin |  | #6 |
| Water bubbling |  | #4 |
| Water draining |  | #4 |
| Water dripping |  | #4 |
| Whip |  | #8 |
| Whistling tune |  | #6 |
| Whistle |  | #1 |
| Wind |  | #4 |
| Wind chimes 1 |  | #6 |
| Wind chimes 2 |  | #6 |
| Wolf |  | #2 |
| Woodpecker |  | #3 |
| Yawn |  | #1 |
| Zipper |  | #3 |
