# Supplementary material for: A Mathematical Approach to Correlating Objective Spectro-Temporal Features of Non-linguistic Sounds With Their Subjective Perceptions in Humans
Source: Front Neurosci. 2019 Jul 31;13:794. doi: 10.3389/fnins.2019.00794 (PMC6685481; doi:10.3389/fnins.2019.00794)
Supplement: Supplementary file 2 [file Table_2.DOCX]

SUPPLEMENTARY MATERIAL 2

The measures which were selected for our study where chosen for differing reasons; these reasons and their method of calculation are described for each selected measure below:

### a) Sample entropy

Entropic measures were considered due to their established use in implying the complexity of biological signals such as electroencephalography (Rezek & Roberts, 1998; Bruhn et al., 2000), respiratory motion (Burioko et al., 2003), and heart rate (Lake et al., 2002). Entropic measures are based on a number of different theories, the two most important of which are information theory (how much unique information is contained in a signal; Hartley, 1928; Shannon, 1948; Kolmogorov, 1968) and chaos theory (how perceivably unpredictable sequential information appears; Lorenz, 1963; Mandlebrot, 1963). We therefore included one major entropic measure from each of these two theoretical perspectives of complexity.

From information theory-based entropic measures, we chose sample entropy (Richman & Moorman, 2000; Lake et al., 2002) as it is based on the well-known approximate entropy measure (Pincus, 1991; Rezek & Roberts, 1998; Bhattacharya, 2000; Burioka et al. 2003) but lacks its self-matching bias (Richman & Moorman, 2000). It estimates entropy as the likelihood of two identical strings of some length being similar for the next number in the sequence, thereby identifying the amount of repeatable or non-unique information. An added option to sample entropy was use of multi-scale and multi-resolution techniques (Costa et al. 2003; Costa et al. 2005; Torres & Camero, 2000), which would help avoid oversampling or signal artefacts. However, because our signals (NLSs) had been vetted for artefacts, i.e., they were high-quality recordings, and are relatively brief in duration, the option to conduct our sample entropy analyses including these techniques was considered unnecessary.

Therefore, to calculate the sample entropy (Richman & Moorman, 2000) of a NLS, we consider its waveform which has a constant sampling time to have a length of $N$in a single embedding dimension of $m=2$, so as to give a dataset of $N=\{x_{1},x_{2},x_{3},\ldots,x_{N}\}$. A template vector was created with a length of $m$ to map $X_{m}\left( i \right)=\{x_{i},x_{i+1},x_{i+2},\ldots,x_{i+m-1}\}$, as well as a distance function to determine distance within the vector space, e.g. Chebyshev distance $d[x_{m}\left( i \right),x_{m}\left( j \right)]$ where $(i\neq j)$. Next, we determined a standard tolerance $r$ of $0.2 \times std\left( m \right)$, where $std\left( m \right)$ was the standard deviation of $m$ from $1\to N$. Given $r$, we then counted the template vector pairs of length $m$ (which we call $B$) and $m+1$ (which we call $A$) wherever $d\left[ x_{m}\left( i \right),x_{m}\left( j \right) \right]<r$. We then calculated the sample entropy as $SampEn=-log\frac{A}{B}$, where the higher a value, the more unique information present throughout the NLS and vice-versa.

### b) Permutation entropy

For an entropic measure based on chaos theory, we chose permutation entropy (Bandt & Pompe, 2002) as one of the best recognised (Riedl et al., 2012; Zanin et al., 2013) in this domain. It calculates the distribution entropy of permutations of small bins of sequential numbers within the dataset; again with regards to using multi-scale and multi-resolution techniques (Costa et al. 2003; Costa et al. 2005; Torres & Camero, 2000), as our signals were high-quality and brief recordings, we considered these techniques unnecessary.

To calculate the permutation entropy (Bandt & Pompe, 2002) of a NLS which has a constant sampling time and a length of $n$, we constructed an embedding dimension of $D=4$ at each time-point $s$ of the $D$-th subsequent values in the waveform $X=(x_{t}:1=1,\ldots,n\}$ but with a given time delay of $\tau=50$ (in data-points), i.e. $s\mapsto(x_{s+\tau+1},x_{s+\tau+2},x_{s+\tau+3},x_{s+\tau+4})$. An ordinal pattern was then assigned to this created vector as the permutation $\pi=(r_{0}r_{1}r_{2}r_{3})$ of $(01\ldots D-1)$, ensuring that $x_{s+r0}\leq x_{s+r1}\leq x_{s+r2}\leq x_{s+r3}$. We then determined the permutation entropy by applying the Shannon entropy (Shannon, 1948) given a probability distribution of $\prod$for the entire series and considering the frequencies of possible permutation patterns $i$ as elements thereof for $i=1,\ldots,D!$ and defining $PermEn=-\sum_{i=1}^{D!} \pi_{i}{log}_{2}\pi_{i}$.

### c) Lempel-Ziv algorithmic complexity

Another approach to complexity is algorithmic or computational complexity theory, which details how much the original information can be condensed into a computationally-efficient representation, as in an algorithm. Measures based on this theory, such as state machines, have been used in birdsong research with some success (Sasahara & Ikegami, 2004) but this particular measure requires that signals be highly repetitive and so our dataset was not amenable to this analysis. Another measure based on this theory, also relating to complexity, is measurement of hidden Markov model states (Gunther, 1994). However this is not always considered an explicit measurement of complexity and is more often used for classification (Shalizi et al. 2001; Shalizi et al. 2002; Buchler et al. 2005).

One of the most popular and successful measures based on algorithmic or computational complexity theory has been the Lempel-Ziv (LZ) algorithmic complexity measure (Khalatur et al. 2003; Szczepanski et al. 2003; Watanabe et al. 2003; Huang et al. 2003), originally designed as a lossless digital compression algorithm (Ziv J & Lempel A, 1978). It first reduces the complex signal into a list of symbols, and for physiological data binary sequences are sufficient for this purpose (Xu et al., 1997; Radhakrishnan & Gangadhar, 1998; Zhang et al., 2000; Wu & Zu, 2001; Zhang et al., 2001). Then, moving from left to right along the signal, counting is done of how many unique instances of consecutive symbols (or, in our base, ones and zeros) exist in the entire signal. The signal is then normalised according to its length. In the absence of hard-and-fast rules regarding the method of reducing a complex signal to a binary sequence, we opted to use some of the most common methods, including: (i) average binary: a one is assigned to any data-point above the average within the dataset, and the remainder are assigned a zero; (ii) modified zone binary: any data-point outside of the 'modified zone' (the average within the dataset ± the standard deviation) is assigned a one, and the remainder are assigned zero; and (iii) differential binary: where a data-point is positive with respect to that which was immediately before it, a one is assigned (including the first data-point), and where it is negative a zero is assigned.

Having reduced the signal to a binary form using the three abovementioned methods, we then calculated the LZ algorithmic complexity (Aboy et al., 2006) for each of these three sequences separately for each NLS' waveform. For each sequence $P$, subsequences $S$ and $Q$ were created and the last character deleted $\pi$ from $Q$, making $SQ\pi$ the new total sequence. We assigned $v(SQ\pi)$ as the 'vocabulary' of unique subsequences of $SQ\pi$ and began counting the unique subsequences at $c\left( n \right)=1$ where $S=s\left( 1 \right)$ and $Q=s(2)$. This continued as $S=s\left( 1 \right),s\left( 2 \right),\ldots,s(r)$ and $Q=s\left( r+1 \right)$ to give $SQ\pi=s\left( 1 \right),s\left( 2 \right),\ldots,s(r)$, except where $Q$ was already found in the vocabulary $v(SQ\pi)$. Where it was found, $Q$ was increased to be $Q=s\left( r+1 \right),s\left( r+2 \right),\ldots,s\left( r+i \right)$, i.e. until it was not longer a subsequence of $SQ\pi=s\left( 1 \right),s\left( 2 \right),\ldots,s(r+i-1)$. Each time a new subsequence was found, $c\left( n \right)$ was increased by one. After exhausting a subsequence, the subsequences were refreshed as $S=s\left( 1 \right),s\left( 2 \right),\ldots,s(r+i)$ and $Q=s\left( r+i+1 \right)$ until $Q$ was the final digit in the sequence (the penultimate of the original sequence $P$). To normalise this complexity measure $c\left( n \right)$ of $P$, we normalised $c\left( n \right)$according the length $n$ of $P$ given the number of used symbols $\alpha=2$ and determining the upper-bound of $c\left( n \right)$ as $b\left( n \right)=\frac{n}{{log}_{2}(n)}$ , defining the normalised LZ complexity $C\left( n \right)$ as $C\left( n \right)=\frac{c(n)}{b(n)}$.

### d) Duration

Sound duration is well established as an important feature of an NLS event (Ballas, 1993; Gygi et al. 2007) and can impact on its identification success (Ballas, 1993; Ballas et al., 1986; De Lucia et al., 2012) and in objective classification (Gygi et al. 2007; Maher & Studniarz, 2012). As one important measure in our study was identification success of NLSs, we included sound duration as an important measure. However, it did not require a formal calculation for our database as the NLSs' durations were already associated for each individual sound.

### e) Peaks-related measures

Peaks-related measures has been less widely used in NLS research but was used in an important, comprehensive previous study of NLSs (Gygi et al. 2007), and inclusion of this measure would allow our results to more easily supplement or be compared with this previous study. There are multiple peak-related measures all of which rely on identifying peaks in a sound's waveform. Because a 'peak' could be highly specific to a particular sound, i.e. there may be moments of relative silence and relative loudness, we chose to first identify the range of data-points within a sound's waveform, and call any data-point a 'peak' if its amplitude was $>0.8\times range$ where the $range$ was the overall amplitude range within the NLS. Given the number of peaks and their relative amplitudes (where 1 is the greatest amplitude) for a NLS, three measures were derived: (i) the number of peaks per second; (ii) the mean relative amplitude of all the peaks; and (iii) the standard deviation of the relative amplitude of all the peaks.

### f) Fractal dimension estimations

Since we were interested in the complexity of NLS', any measures which inferred or estimated the complexity of a biological signal were considered for inclusion in our study. In addition to the most common of these, entropic measures which were discussed earlier, another very well-known measure is fractal dimension (FD) estimation (Spasic et al., 2005; Shibayama, 2006; Raghavendra & Dutt, 2010).

Formally, all geometric objects have both a topological and fractal dimension; however the former is always an integer whereas the latter may be an integer or a non-integer. We can define the topological dimension of an object as the number of independent directions it is possible to move within that object, e.g. on a curve we can only move in one direction or the reverse ∴ topological dimension of a curve = 1. Whereas the definition of a fractal dimension is much more complex (Mandelbrot, 1983; Barnsley, 1993) but essentially is a measure of self-similarity within a geometric object after magnification. A well-known fractal (object which has a fractal dimension > topological dimension) is the Koch snowflake (Koch, 1904), which is a curve of infinite length and a topological dimension of 1, however its fractal dimension is $\frac{ln 4}{ln 3}$ = 1.26186…, i.e. it is self-similar to the extent of being complex beyond its topological dimension.

Because NLSs are real signals - i.e. unlike the Koch snowflake, they have a finite length - we are assuming fractal properties are present within them and then estimating what their fractal dimension could be if they were of an infinite length. Of the methods available for fractal dimension estimation of one-dimensional signals, some have analysed artificial signals (Raghavendra and Dutt, 2010) in order to compare their estimations with parametric fractal signals which have known theoretical fractal dimensions. This has enabled such methods to be tested for their accuracy. Of these methods, the Higuchi method (Higuchi, 1998), which was originally designed to analyse magnetic field data, has become one of the most commonly used for one-dimensional signals. However, a method called the Normalised Length Density (NLD) has also been developed for very short samples or epochs, especially useful where there are 100 data-points or less (Kalauzi et al. 2009). Although both of these methods of FD estimation - Higuchi and NLD - attempt to estimate the same feature of a signal, they do so in very different ways. Because of this difference, we chose to include both methods in our study.

To calculate the FD estimate using the Higuchi method (Higuchi, 1998), we let $X$ be the one-dimensional time series of the waveform for a given NLS $X = x[1], x[2],\ldots,x[N],$ where $N$ is the final sample in the series. We then defined a new time series as

$$X_{k}^{m}=\{x\left[ m \right], x\left[ m+k \right],x\left[ m+2k \right],\ldots,$$

$$x\left. \left[ m+int\left( \frac{N-m}{k} \right)\times k \right] \right\}$$

where $k$ was the sampling frequency and $m$ was the initial time point in the series. We then found the length of the new time series $X_{k}^{m}$ by

$$L\left( m,k \right)= \frac{\left\{ \left( \sum_{i=1}^{int\left( \frac{N-m}{k} \right)} \left| x\left[ m+ik \right]-x[m+(i-1)\times k] \right| \right)\left[ \frac{N-1}{int \left( \frac{N-m}{k} \right)}\times k \right] \right\}}{k}$$

where $N$ is the length of the original time series $X$. The length for the time interval $k$ was then defined as

$L\left( k \right)= \frac{1}{k}\times\sum_{m=1}^{k} L(m,k)$.

To calculate the actual estimate, we used a double-logarithmic scale to plot $L(k)$ against $\frac{1}{k}$, with $k=1,2,\ldots,k_{max}$, where the slope $\left\{ ln[L\left( k \right)],ln\left( \frac{1}{k} \right) \right\}$ was equal to the fractal dimension estimate of the original one-dimensional time series $X$ of the NLS. For this study a value of $k_{max}=10$ was chosen as after testing $k_{max}=2,3,\ldots,50$ it gave us the greatest dynamic range (Spasic et al. 2005) within our dataset of NLSs.

The second method, NLD (Kalauzi et al. 2009), is much simpler than the Higuchi method. We let $y$ be the amplitude normalized one-dimensional time series of the waveform for a given NLS $y = y(1), y(2),\ldots,y(N),$ where $N$ was the final sample in the series. We then defined the $NLD$ as

$$NLD= \frac{1}{N}\sum_{i=2}^{N} \left| y_{n}\left( i \right)-y_{n}(i-1) \right|$$

where $y_{n}\left( i \right)$ was the $i$th sample in the time series. We then used the power model (Kalauzi et al. 2009) to convert the $NLD$ to an FD estimate

$$FD={a(NLD-{NLD}_{0})}^{k}$$

where $a=1.9079$, ${NLD}_{0}=0.097178$ and $k=0.18383$.

### g) Mean spectral centroid

The mean spectral centroid, similarly to duration, is a well-reported, important feature of NLSs in terms of objective classification (Shao et al. 2003; Gygi et al. 2007; Maher & Studniarz, 2012). It has also been aligned with subjective dimensions of musical instruments (Grey & Grodon, 1978). For these reasons, we chose to include it in our study.

Calculation of the mean spectral centroid was based on the fast Fourier transform (FFT) of the NLS and given as:

$$\omega_{C}\frac{\int_{0}^{\omega_{0}} \omega|F\left( \omega\right)|^{2}d\omega}{\int_{0}^{\omega_{0}} |F\left( \omega\right)|^{2}d\omega}$$

where $\omega_{0}$ is half the sampling frequency of the NLS.

### h) Root mean square of frequency ranges

The root mean square (RMS) of frequency ranges was employed by Gygi et al. (2007) in their objective classification of NLSs, and so inclusion of this measure in our study would help provide a comparison. High power at certain low- and mid-range frequencies have also been heavily implicated with the perception of unpleasantness (Halpern et al., 1986; Kumar et al., 2008; Reuter & Oehler, 2011), however these studies have used varying techniques to reach their conclusions.

As conducted in the Gygi et al. (2007) study, we filtered NLSs to isolate certain frequency ranges and then calculated the RMS of those filtered signals. It is important at this point to remember that all NLSs in our database had been normalised for amplitude (RMS) already, so comparing the RMSs of different NLSs was appropriate. However, unlike the Gygi et al. (2007) study, our study rests in the context of a larger study concerned with hearing tests. Because of this, we chose frequency ranges based upon the frequencies commonly tested using audiometry (WHO, 1991). We therefore chose the following frequency ranges to isolate via filtering: 250-2500Hz, 500-1000Hz, 1000-2000Hz, and 2000-4000Hz.

### i) Harmonics-to-noise ratio

Previous studies have defined harmonicity as an important feature of NLSs (Gygi et al., 2007; Lewis et al., 2009); hence, we included one of the most recognised harmonicity measures, the harmonics-to-noise ratio (HNR). This measure was originally developed as a psychophysical rating of the hoarseness of human speech (Yumoto et al. 1982). Later studies have used it in the study of NLS features, e.g. correlating it and other features with functional magnetic resonance imaging of brain areas (Lewis et al. 2009). The HNR quantifies the relative power of harmonic contents of a sound as the energy of its periodic contents in comparison to its non-periodic contents (Boersma, 1993). The value of zero represents equal contents, a negative value represents a greater non-periodic contents than periodic contents (e.g. white noise), and vice-versa (e.g. a wolf howl).

To calculate the HNR, we let $f(t)$ be the original one-dimensional waveform for a given NLS and $f_{i}(\tau)$ be the waves for each pitch period $\tau$. We then defined the HNR as:

$$\frac{H}{N}= \frac{n \int_{0}^{T} f_{A}^{2}\left( \tau\right)d\tau}{\sum_{i=1}^{n} \int_{0}^{T_{i}}{[f_{i}\left( \tau\right)-f_{A}\left( \tau\right)]}^{2}d\tau}$$

where $n$ is the number of pitch periods and $d$ is the duration of the pitch period.

### j) Spectral flatness measure

The spectral flatness measure (SFM), mostly used in phonetics research (Boersma, 2001), was included due to its unique status as an entropy-related measure in the spectral domain and its use as such in previous sound research (Jayant and Noll, 1984; Boersma, 2001) in contrast to most entropy-based measures, e.g. permutation entropy (Riedl et al., 2012; Zanin et al., 2013), which measure in the time domain and are not widely used in sound research. The SFM measures the degree of uniformity within a signal’s power spectrum (Jayant and Noll, 1984), with white noise = 1 (chaos) and a pure tone = 0 (complete order). However to expand its dynamic range, it is measured on a logarithmic scale so that white noise = log1 = 0 and a pure tone = log0 = -∞. This results in large negative numbers for simple sounds and numbers approaching zero for complex sounds.

To calculate the SFM, we let $s(t)$ be the FFT of the time-series of a NLS and defined the SFM as:

$$SFM=log\frac{\left[ \prod_{f=1}^{N} S(f) \right]^{\frac{1}{N}}}{\left( \frac{1}{N} \right)\sum_{f=1}^{N} S(f)}$$

where $N$ was the number of FFT points and $S(f)$ was the amplitude (Hz) at each point. The result was then logged (1) to expand the dynamic range.

#### k) Spectral structure variability or index

Using the derived SFM from a signal, we can then calculate the spectral structure variability (SSV) or index (SSI) as the variability of the SFM over time. It is conceptually similar to the way a spectrogram shows the variability of Fourier transforms over time. This measure has been used previously in the analysis of NLSs (Reddy et al. 2009; Singh, 2011) and human vocalisations (Talkington et al. 2012). It has even been suggested as a direct measure of NLS complexity (Singh, 2011); hence it was considered suitable for inclusion in our study.

The SSI gives a pure number, where if SSI<1 the sound has similar spectral dynamics (simple) and if SSI>1 the sound has dissimilar spectral dynamics (complex). To calculate it, we let $s(t)$ be the FFT of the time-series of a NLS and defined the SSI as:

$$SSI= \sum\frac{\left[ SFM\left( t \right)-\frac{\sum SFM(t)}{N} \right]^{2}}{N}$$

where $N$ is the number of time frames of:

$$SFM\left( t \right)=log\frac{\left[ \prod_{i=1}^{N} S(t,f) \right]^{\frac{1}{N}}}{\left( \frac{1}{N} \right)\sum_{i=1}^{N} S(t,f)}$$

and where $S(t,f)$ is the power with that frequency of that time-frame.
